# Ventriculoperitoneal Shunt-Associated Cerebrospinal Fluid Pseudocyst Presenting as Abdominal Distension and Pain in an Adult Female Patient: A Case Report

**DOI:** 10.7759/cureus.89277

**Published:** 2025-08-03

**Authors:** Pokhraj P Suthar, Sumeet Virmani

**Affiliations:** 1 Department of Diagnostic Radiology and Nuclear Medicine, Rush University Medical Center, Chicago, USA

**Keywords:** csf, ct, management, ultrasound, ventriculoperitoneal shunt

## Abstract

Ventriculoperitoneal (VP) shunt placement is a common and effective intervention for managing hydrocephalus. While generally successful, this procedure can be associated with rare but serious complications, including cerebrospinal fluid (CSF) pseudocyst formation. These loculated, epithelial-free fluid collections typically form around the distal catheter in the peritoneal cavity and are more commonly seen in pediatric patients. In adults, their presentation may be delayed and often nonspecific, leading to diagnostic challenges.

We report the case of a 32-year-old female patient with a history of congenital hydrocephalus and multiple VP shunt revisions, presenting with worsening abdominal pain, distension, and gastrointestinal symptoms. Imaging revealed a large, loculated intraperitoneal fluid collection encapsulating the distal tip of the VP shunt, consistent with a CSF pseudocyst. Ultrasound-guided paracentesis was performed. The patient showed symptomatic improvement following fluid drainage and conservative management. CSF pseudocyst should be considered in the differential diagnosis of abdominal complaints in adults with a history of VP shunt placement, regardless of the time elapsed since the initial surgery. Prompt imaging and multidisciplinary care are essential for diagnosis and management to prevent complications and recurrence.

## Introduction

Ventriculoperitoneal (VP) shunts are a widely used and effective treatment for hydrocephalus, allowing diversion of cerebrospinal fluid (CSF) from the brain to the peritoneal cavity [1,2]. Despite their success, VP shunts are associated with a variety of complications, including obstruction, infection, and abdominal issues such as CSF pseudocyst formation [3-6]. A CSF pseudocyst is an uncommon but significant complication that typically develops around the distal catheter tip and presents as a loculated fluid collection without an epithelial lining. While more frequently observed in pediatric populations, pseudocysts can also occur in adults, where they often present with vague, nonspecific symptoms like abdominal pain, distension, and gastrointestinal discomfort [3]. In adult patients, CSF pseudocysts can mimic other intraabdominal pathologies, making diagnosis challenging, particularly when there is a remote history of shunt placement. Imaging studies such as ultrasound and CT scans are critical for diagnosis, and paracentesis can aid both in symptom relief and evaluation of infection [7]. Management usually involves surgical revision of the shunt and, when needed, antimicrobial therapy [8]. This case highlights the importance of considering CSF pseudocyst in the differential diagnosis of abdominal symptoms in adults with a history of VP shunt placement, even many years after initial surgery.

## Case presentation

A 32-year-old female patient presented to the emergency department with a 12-hour history of worsening, crampy abdominal pain radiating bilaterally to the lower back. She reported progressive abdominal distension and constipation over the preceding two weeks. At the time of initial evaluation, she denied nausea, vomiting, diarrhea, fever, urinary symptoms, or hematochezia. However, during her clinical assessment, she developed new-onset nausea with associated vomiting (approximately four episodes per day), as well as headache and blurred vision.

Her past medical history was notable for congenital syphilis-associated hydrocephalus requiring VP shunt placement in childhood. She had undergone three shunt revisions, the most recent in 2011. Additional medical history included asthma, bipolar disorder, major depressive disorder, gastroesophageal reflux disease (GERD), and opioid use disorder. She was currently prescribed buprenorphine (Subutex) 8 mg daily and as-needed clonidine.

On presentation, the patient was hemodynamically stable. Her blood pressure was 121/81 mmHg, heart rate of 88 beats per minute, respiratory rate of 18 breaths per minute, temperature of 98.1°F (36.7°C), and oxygen saturation of 100% on room air.

The patient appeared in moderate distress due to abdominal pain and headache and was actively vomiting. She was alert and oriented but minimally cooperative due to discomfort. Her abdominal examination revealed significant distension with diffuse tenderness and hypoactive bowel sounds. No peritoneal signs such as rebound tenderness, rigidity, or guarding were elicited. Neurologically, the patient was alert and oriented to person, place, and time, with no focal deficits on examination.

Initial laboratory investigations revealed a normal white blood cell count (5.16 K/uL) and mild anemia (hemoglobin 11.9 g/dL). The remainder of the complete blood count and the basic metabolic panel were within normal limits (Table 1). No laboratory evidence of systemic infection or inflammatory response was identified. 

**Table 1:** Laboratory values MCV: mean corpuscular hemoglobin; MCH: mean corpuscular hemoglobin; MCHC: mean corpuscular hemoglobin concentration; RDW: red cell distribution width; ALT: alanine aminotransferase; AST: aspartate aminotransferase

Test	Result	Reference range
White blood count (WBC)	5.16	4.00-10.00 K/uL
Red blood count (RBC)	4.51	4.00-5.20 M/uL
Hemoglobin	11.9	12.0-16.0 g/dL
Hematocrit	38.5	37.0-47.0%
MCV	85.4	82.0-103.0 fL
MCH	26.4	26.0-34.0 pg
MCHC	30.9	30.0-37.0 g/dL
RDW	13.1	11.5-14.5%
Platelet count	308	150-399 K/uL
Auto neutrophil #	3.49	1.84-7.80 K/uL
nRBC/100 WBCs	0	0/100 WBC
Neutrophils %	67.6	46.0-78.0%
Lymphocyte %	24.4	18.0-52.0%
Monocyte %	6.0	3.0-10.0%
Eosinophil %	1.6	0.0-6.0%
Basophil %	0.2	0.0-3.0%
Neutrophil #	3.49	1.84-7.80 K/uL
Lymphocyte #	1.26	0.72-5.20 K/uL
Monocyte #	0.31	0.12-1.00 K/uL
Eosinophil #	0.08	0.00-0.60 K/uL
Basophil #	0.01	0.00-0.30 K/uL
Immature gran #	0.01	0.00-0.15 K/uL
Immature gran %	0.2	0.0-1.5%
Sodium	135	137-147 mmol/L
Potassium	4.5	3.4-5.3 mmol/L
Chloride	102	99-108 mmol/L
CO2 total	25	22-29 mmol/L
Anion gap	13	10-20
Blood urea nitrogen (BUN)	8	8-21 mg/dL
Creatinine	0.74	0.65-1.00 mg/dL
Glucose	99	60-99 mg/dL
Calcium	9.6	8.7-10.7 mg/dL
Total protein	7.7	6.0-8.2 g/dL
Albumin	3.5	3.5-5.0 g/dL
Bilirubin total	0.7	0.2-1.3 mg/dL
Alkaline phosphatase	54	30-125 U/L
ALT	7	0-40 U/L
AST	Not listed	3-44 U/L
Lipase	15	10-52 U/L
C-reactive protein (CRP)	9.6	0.0-8.0 mg/L
ESR-Westergren	20	0-27 mm/hr

**Figure 1:**
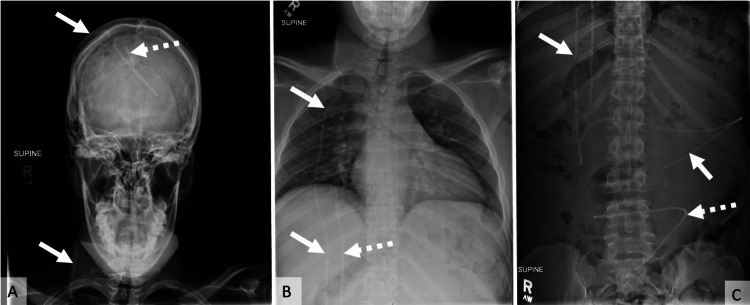
X-ray shunt series (A) Skull, (B) neck and chest, and (C) abdomen demonstrate an intact shunt catheter (solid white arrows) with a few abandoned catheters (dashed white arrows)

**Figure 2:**
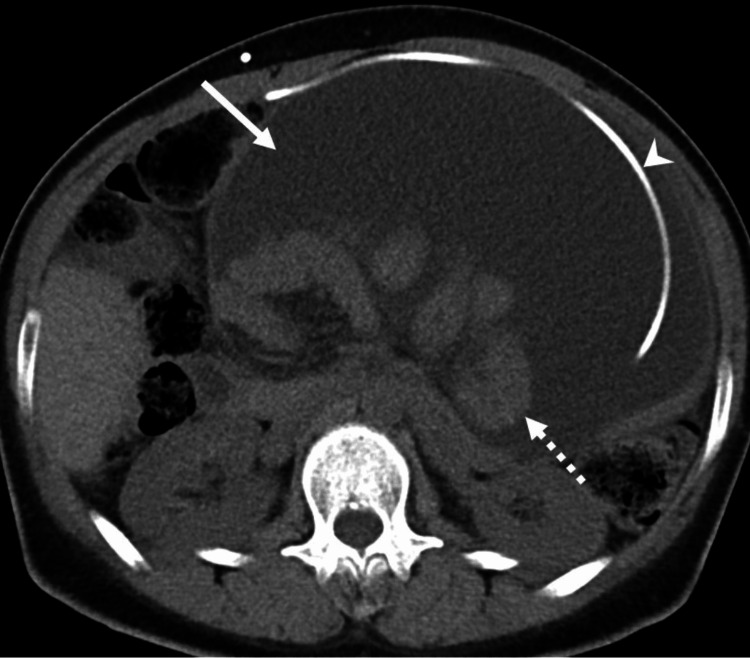
CT scan without contrast CT: computed tomography; VP: ventriculoperitoneal; CSF: cerebrospinal fluid Revealed a large, loculated intraperitoneal fluid collection (solid white arrow) with the VP shunt catheter (white arrowhead) situated within, indicative of a CSF peritoneal pseudocyst. Nondilated small bowel loops were also noted within this collection (dashed arrow)

A VP shunt series performed on admission demonstrated an intact shunt catheter with a few abandoned catheters (Figure 1). A noncontrast computed tomography (CT) scan of the abdomen confirmed the presence of a large, loculated intraperitoneal fluid collection encapsulating the distal end of the VP shunt catheter. Nondilated small bowel loops were observed within the fluid, consistent with a CSF peritoneal pseudocyst (Figure 2).

**Figure 3:**
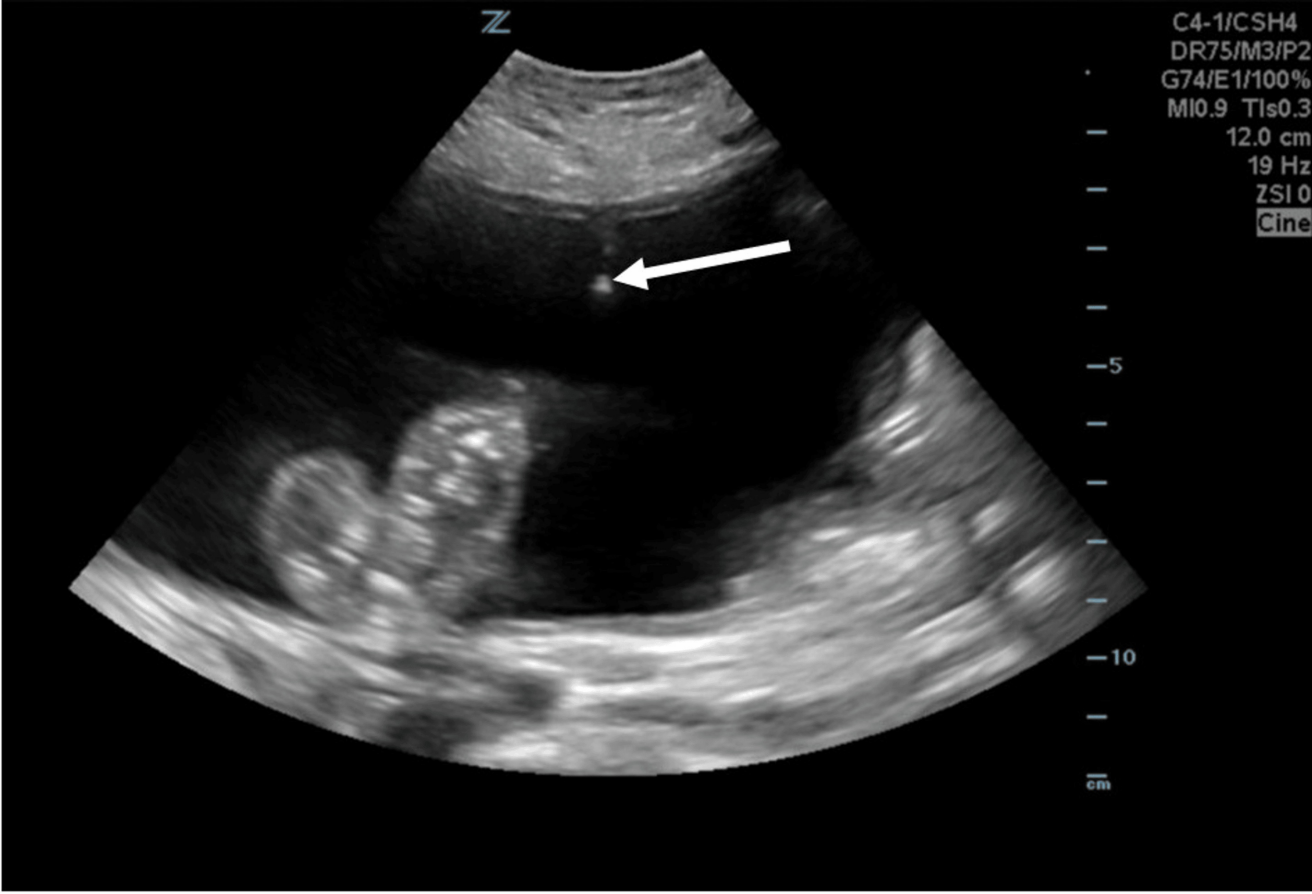
Ultrasound-guided paracentesis Ultrasound-guided paracentesis with the needle tip (white arrow) of the paracentesis needle

An ultrasound-guided paracentesis was subsequently performed, yielding approximately 3 liters of clear ascitic fluid (Figure 3). Microbiological analysis of the aspirate was negative for bacterial and fungal growth. No organisms were visualized on Gram stain. Additionally, no acid-fast bacilli (AFB) were seen on fluorochrome staining, and no AFB were isolated after eight weeks of incubation. 

The patient was empirically initiated on broad-spectrum antibiotics, including cefepime, metronidazole, and vancomycin. These were discontinued once the culture results returned negative. Following fluid drainage, the patient reported marked improvement in abdominal symptoms. The Neurosurgery and General Surgery services were consulted and engaged in a detailed discussion regarding potential surgical management options. However, the patient elected not to proceed with the proposed intervention at this time, expressing a desire to obtain a second opinion from another neurosurgical team.

## Discussion

Hydrocephalus is a chronic neurological condition characterized by the abnormal accumulation of CSF within the ventricles of the brain, often requiring long-term CSF diversion through the use of a VP shunt [1,2]. VP shunt placement is a widely used and effective procedure, particularly in both pediatric and adult patients, to relieve increased intracranial pressure [1,2]. Despite its clinical utility, VP shunting is associated with several potential complications, including infection, obstruction, overdrainage, and mechanical failure [9,10]. Among the less common but clinically significant complications is the formation of an abdominal CSF pseudocyst [3].

A CSF pseudocyst is a loculated fluid collection typically forming around the distal tip of the VP shunt catheter within the peritoneal cavity. Unlike true cysts, these lesions lack an epithelial lining and are composed of fibrous tissue, often forming in response to chronic inflammation, repeated shunt revisions, or low-grade infection. Though rare, pseudocysts may arise months to years after the initial VP shunt placement [3]. They occur more frequently in pediatric patients, yet adult presentations, although less common, can pose substantial diagnostic challenges due to their nonspecific and often misleading clinical features [3]. Patients with CSF pseudocysts may present with a wide range of symptoms, including abdominal pain, nausea, vomiting, bloating, constipation, and signs of increased intracranial pressure if the shunt becomes dysfunctional [3]. Notably, while pediatric cases often present with classic signs of shunt malfunction such as vomiting and headache, adult presentations more frequently involve isolated abdominal symptoms, making the diagnosis less straightforward [11]. As a result, CSF pseudocysts can be misdiagnosed as intraabdominal abscesses, ascites, or neoplastic masses if a high index of suspicion is not maintained, particularly in those with remote histories of shunt placement [11].

The diagnosis is typically confirmed through imaging, with abdominal ultrasonography being a sensitive and noninvasive first-line modality [10,12]. While ultrasound is sensitive in detecting intraabdominal fluid collections, the wall of a pseudocyst may not be well-visualized due to imaging limitations and acoustic incoherence. In such cases, CT provides superior anatomical detail and is more helpful in delineating the extent and morphology of the pseudocyst. CT can further delineate the extent and characteristics of the fluid collection and help evaluate for associated complications [10]. In some cases, paracentesis may be necessary for diagnostic clarification and symptom relief. Cultures of aspirated fluid help guide antibiotic therapy when infection is suspected. Treatment of CSF pseudocysts generally involves surgical intervention. This may include externalization of the distal catheter, relocation of the peritoneal catheter to a different quadrant, or conversion to an alternative drainage route such as a ventriculoatrial or ventriculopleural shunt. When infection is identified, appropriate antimicrobial therapy is essential, and recurrence is uncommon with adequate treatment and resolution of the underlying cause. Risk factors for pseudocyst formation include prior shunt revisions, abdominal infections, peritonitis, and immunocompromised states [13].

Although the majority of documented pseudocysts are nonhemorrhagic, rare cases of hemorrhagic pseudocysts have been reported. Wang et al. described a case of hemorrhagic abdominal pseudocyst following VP shunt placement, underscoring the potential for atypical presentations and the need for clinical vigilance [14]. A case reported by Yim et al. demonstrated the development of infection within a CSF pseudocyst after initial symptomatic improvement, highlighting the potential for delayed infectious complications in patients with VP shunts [15]. The recurrence rate of CSF pseudocysts is higher following simple drainage, emphasizing the need for definitive management to prevent relapse.

This case report presents a rare instance of a large, symptomatic CSF pseudocyst in a 32-year-old adult female patient with a remote history of congenital hydrocephalus and multiple VP shunt revisions. The patient presented with abdominal pain, distension, and gastrointestinal symptoms but was ultimately found to have a CSF pseudocyst surrounding the distal catheter tip. This case highlights the importance of considering VP shunt-related complications in adults with relevant surgical histories, even many years after initial placement, and the role of cross-sectional imaging and multidisciplinary care in diagnosis and management.

## Conclusions

CSF pseudocysts are a rare but clinically significant complication of VP shunt systems, particularly in adults with remote shunt placement. Due to their often nonspecific presentation, typically limited to abdominal symptoms, these cases require a high index of suspicion, especially in patients with a known history of hydrocephalus or prior shunt revisions.

Early recognition using appropriate imaging, along with coordinated multidisciplinary management involving neurosurgery and interventional radiology, is essential for accurate diagnosis and timely treatment. The general approach included abdominal ultrasound for patients presenting with symptoms of shunt malfunction without an identifiable alternative cause. When a pseudocyst was identified, fluid cultures were obtained from both the shunt system and the pseudocyst, and empirical intravenous antibiotics were initiated. Subsequent management, including laparoscopic repositioning or shunt externalization, was determined based on clinical course, culture results, inflammatory markers, and findings on follow-up imaging.
